# Functional expression of a blood tolerant laccase in *Pichia pastoris*

**DOI:** 10.1186/1472-6750-13-38

**Published:** 2013-04-30

**Authors:** Diana M Mate, David Gonzalez-Perez, Roman Kittl, Roland Ludwig, Miguel Alcalde

**Affiliations:** 1 Department of Biocatalysis, Institute of Catalysis, CSIC, Madrid 28049, Spain; 2 Department of Food Sciences and Technology, Food Biotechnology Laboratory, BOKU-University of Natural Resources and Life Sciences, Vienna 1190, Austria

**Keywords:** High-redox potential laccase, Functional expression, *Pichia pastoris*, *Saccharomyces cerevisiae*, Directed evolution, Halide inhibition, Hydroxyl inhibition, Blood tolerance

## Abstract

**Background:**

Basidiomycete high-redox potential laccases (HRPLs) working in human physiological fluids (pH 7.4, 150 mM NaCl) arise great interest in the engineering of 3D-nanobiodevices for biomedical uses. In two previous reports, we described the directed evolution of a HRPL from basidiomycete PM1 strain CECT 2971: i) to be expressed in an active, soluble and stable form in *Saccharomyces cerevisiae*, and ii) to be active in human blood. In spite of the fact that *S. cerevisiae* is suited for the directed evolution of HRPLs, the secretion levels obtained in this host are not high enough for further research and exploitation. Thus, the search for an alternative host to over-express the evolved laccases is mandatory.

**Results:**

A blood-active laccase (ChU-B mutant) fused to the native/evolved α-factor prepro-leader was cloned under the control of two different promoters (P_AOX1_ and P_GAP_) and expressed in *Pichia pastoris*. The most active construct, which contained the P_AOX1_ and the evolved prepro-leader, was fermented in a 42-L fed-batch bioreactor yielding production levels of 43 mg/L. The recombinant laccase was purified to homogeneity and thoroughly characterized. As happened in *S. cerevisiae,* the laccase produced by *P. pastoris* presented an extra N-terminal extension (ETEAEF) generated by an alternative processing of the α-factor pro-leader at the Golgi compartment. The laccase mutant secreted by *P. pastoris* showed the same improved properties acquired after several cycles of directed evolution in *S. cerevisiae* for blood-tolerance: a characteristic pH-activity profile shifted to the neutral-basic range and a greatly increased resistance against inhibition by halides. Slight biochemical differences between both expression systems were found in glycosylation, thermostability and turnover numbers.

**Conclusions:**

The tandem-yeast system based on *S. cerevisiae* to perform directed evolution and *P. pastoris* to over-express the evolved laccases constitutes a promising approach for the *in vitro* evolution and production of these enzymes towards different biocatalytic and bioelectrochemical applications.

## Background

Laccases (benzenediol:oxygen oxidoreductase, E.C. 1.10.3.2.) are extracellular, multicopper oxidases widely distributed in fungi, higher plants, bacteria, lichens and insects [1-3]. They contain a T1 copper atom at which the reducing substrate is oxidized and a trinuclear copper cluster at which oxygen is reduced to water [4]. Laccases are able to oxidize a broad range of phenolic and non-phenolic compounds expanding further its broad substrate specificity through the inclusion of redox mediators from natural or artificial sources [5]. The physiological roles of laccases are diverse and depend on their origin. In plants, these enzymes seem to be involved in wound response, fruiting body formation, cell-wall reconstitution and synthesis of lignin [6]. Role attributes for bacterial laccases cover copper homeostasis, morphogenesis and pigmentation of spores to confer resistance to stress factors [2]. In fungi, laccases carry out a variety of physiological roles including morphogenesis, fungal plant-pathogen/host interaction and lignin mineralization [1]. Among fungal laccases, those produced by the basidiomycete white-rot fungi are of great biotechnological interest due to their higher redox potential at the T1 site [7]. Thus, high-redox potential laccases (HRPLs) find applications in the production of second generation biofuels, pulp-kraft biobleaching, bioremediation, organic syntheses and the development of biosensors and miniature biofuel cells for medical uses [8-10].

Over 20 fungal laccases have been heterologously expressed in the yeasts *Pichia pastoris* and *Saccharomyces cerevisiae* for different purposes [11,12]. In general terms, both organisms are suitable for the expression of eukaryotic genes. These hosts are easy to manipulate due to the availability of a large set of molecular biology tools; besides, they have the ability to perform post-translational modifications (disulfide bridge formation, C- and N-terminal processing, glycosylation) readily secreting active enzymes to the culture broth [13]. Particularly, *S. cerevisiae* arise a great interest in synthetic biology and protein engineering by directed evolution [14,15]. With a sophisticated eukaryotic device supported by a high frequency of homologous DNA recombination, the construction of complex metabolic pathways by *in vivo* splicing expression cassettes and/or the directed evolution of cumbersome systems (*e.g.* ligninolytic enzyme consortiums) are simply performed [15,16]. Indeed, the battery of reliable *in vivo* recombination methods based on *S. cerevisiae* physiology make this budding yeast a powerful cell factory for plenty of potential applications [15]. Despite these advantages, the practical use of *S. cerevisiae* in different industrial settings is limited by its rather low secretion levels [11]. Although the methylotrophic yeast *P. pastoris* is not the favorite host for directed evolution experiments (the lack of episomal vectors together with low transformation efficiencies constrain the building of mutant libraries) [17], it does show some attractive features which may complement *S. cerevisiae* in the synthetic evolutionary scenario: specifically, the ability to grow at very high cell densities under the control of strong promoters and secrete high amounts of protein [18]. Even though the expression levels reported for recombinant fungal laccases in these yeasts are diverse (Table 1), overall they are much higher in *P. pastoris*, ranging from 4.9 to 517 mg/L [19-25], than in *S. cerevisiae*, where they vary from 2 to 18 mg/L [26-30].

**Table 1:** **List of fungal laccases heterologously expressed in *P. pastoris *and *S. cerevisiae***

**Heterologous host**	**Laccase source**	**Expression yields (mg/L)**	**Promoter**	**Reference**
*P. pastoris*	*Botrytis aclada*^a^	517	GAP^c^	[19]
*P. pastoris*	*Botrytis aclada*^a^	495	AOX1^d^	[20]
*P. pastoris*	*Pleurotus sajor-caju*^b^	4.9	AOX1^d^	[21]
*P. pastoris*	*Pycnoporus cinnabarinus*^b^	8	AOX1^d^	[22]
*P. pastoris*	*Trametes* sp. 420^b^	136	AOX1^d^	[23]
*P. pastoris*	*Trametes* sp. AH28-2^b^	31.6	AOX1^d^	[24]
*P. pastoris*	*Trametes trogii*^b^	17	AOX1^d^	[25]
*S. cerevisiae*	*Melanocarpus albomyces*^a^	7	GAL1^d^	[26]
*S. cerevisiae*	*Myceliophthora thermophila*^a,*^	18	ADH1^c^	[27]
*S. cerevisiae*	PM1^b,*^	8	GAL1^d^	[28]
*S. cerevisiae*	*Pycnoporus cinnabarinus*^b,*^	2	GAL1^d^	[29]
*S. cerevisiae*	*Trametes* sp. C30^b^	2	GAL10^d^	[30]

^a^Ascomycete; ^b^Basiodiomycete; ^c^Constitutive promoter; ^d^Inducible promoter. ^*^Laccase functional expression achieved by directed evolution.

In a previous work we tackled the directed evolution of the HRPL from the white-rot fungus PM1 strain CECT 2971 to be secreted in *S. cerevisiae* (with levels of ~8 mg/L) [28]. This evolved PM1 laccase was recently tailored to be active in human blood (at pH 7.4 and high NaCl concentration −150 mM-) [31]. HRPLs are strongly inhibited by modest concentrations of OH^-^ and Cl^-^, which tightly bind to the catalytic copper centers interrupting the catalysis. To surpass such inhibition, several rounds of laboratory evolution in combination with semi-rational approaches were carried out using a screening assay based on the biochemical composition of human blood. Here, we describe the cloning and over-expression of this blood tolerant laccase in *P. pastoris*. The recombinant enzyme was tested with different promoters and fermentation conditions. The fermentation of the best construct was scaled up in a 42L-bioreactor to 20L fermentation volume, purified, and biochemically characterized. Laccase properties were compared to those obtained for the same mutant enzyme expressed by *S. cerevisiae*.

## Results and discussion

### Heterologous functional expression of blood tolerant laccases in *P. pastoris*

**Figure 1:**
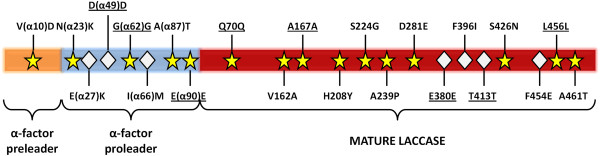
**Mutations in the ChU-B mutant fusion gene.** The α-factor pre-leader is depicted in orange, the α-factor pro-leader in blue, and the mature laccase in red. The 15 mutations accumulated in the directed evolution for functional expression in yeast are represented as yellow stars, while the 7 mutations resulted from the evolutionary process for activity in blood are shown as white diamonds. Silent mutations are underlined.

The departure point of the present study is a thermostable laccase from basidiomycete PM1, which was first subjected to 8 generations of *in vitro* evolution for functional expression in *S. cerevisiae*[28] and thereafter to 4 further cycles of evolution to become active in human blood [31]. The final variant of this process (ChU-B mutant) is formed by the α-factor prepro-leader plus the mature laccase. The ChU-B whole fusion gene harbours 22 mutations (8 silent). Beneficial mutations enhancing functional expression or activity are both located in the signal sequence (5 mutations) and in the mature protein (7 mutations). Besides, the mature protein presents two mutations, F396I and F454E, placed at the second coordination sphere of the T1 Cu, which are responsible for the activity shown in human blood (Figure 1).

**Figure 2:**
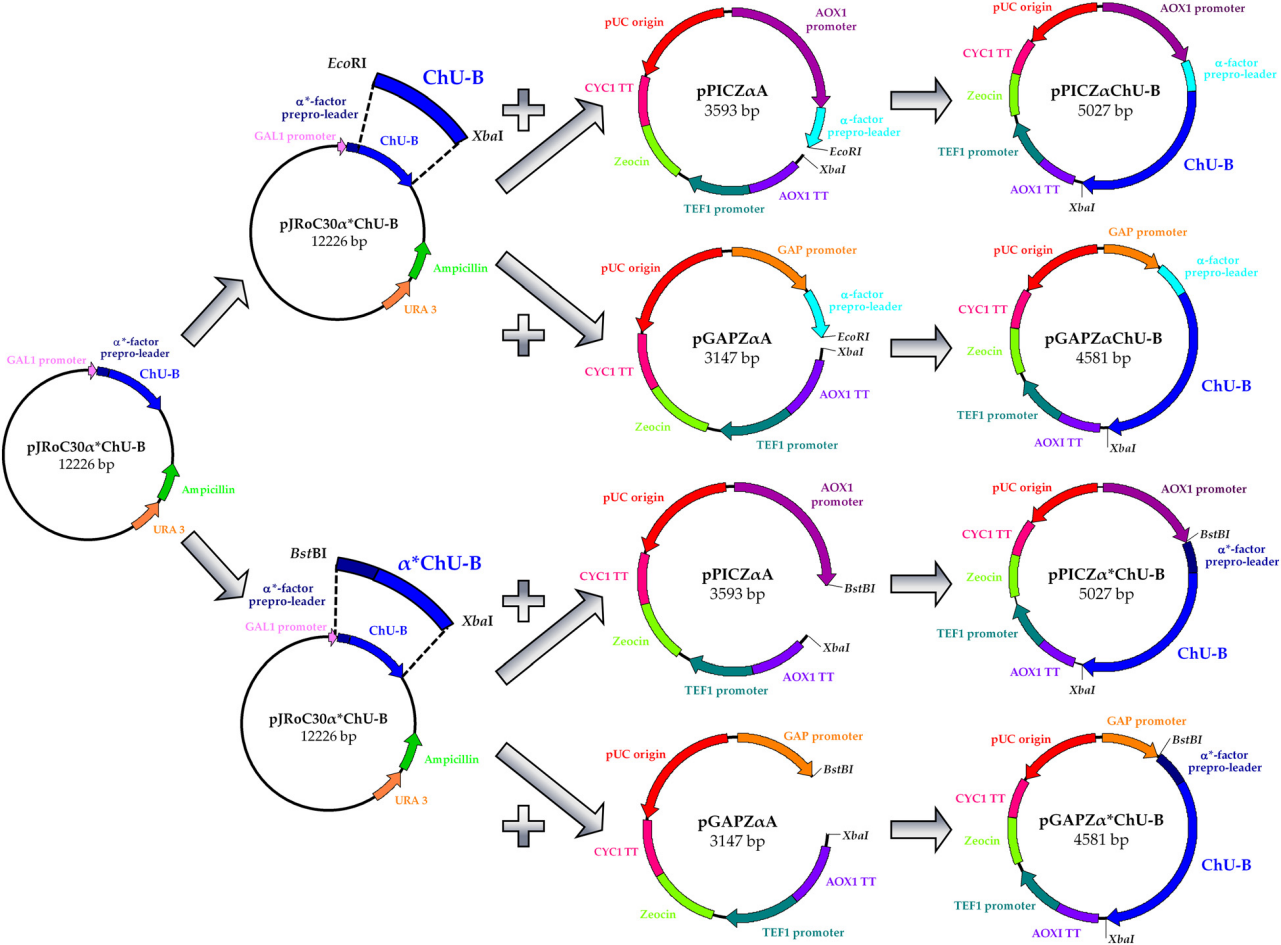
Cloning strategy for the construction of pPICZαChU-B, pGAPZαChU-B, pPICZα*ChU-B and pGAPZα*ChU-B plasmids. The pJRoC30α*ChU-B was used as template to amplify ChU-B with/without the evolved α-factor prepro-leader (α*) from *S. cerevisiae*. See Methods section for details.

**Figure 3:**
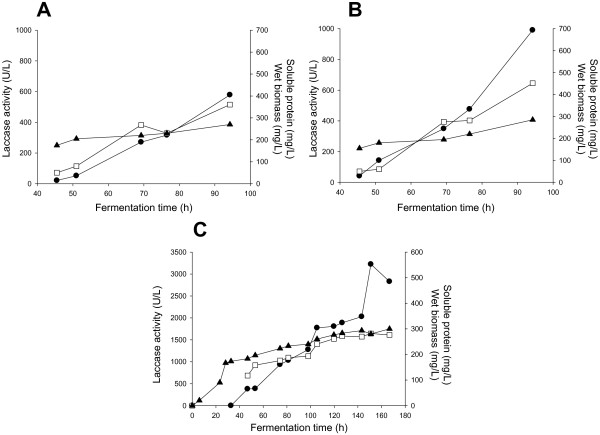
**Laccase expression in *P. pastoris. *** (**A** and **B**) Fermentation in a 500-mL bioreactor of *P. pastoris* transformants expressing the ChU-B mutant joined to the original (**A**) and the evolved (**B**) α-factor prepro-leader under the control of the P_AOX1_. After 22 h of batch phase, glycerol feed was maintained for 5 hours and thereafter the methanol induction started. Methanol was pumped to the culture so that a DO level ~4% was maintained throughout the fermentation. (**C**) 20-L fermentation in a 42-L bioreactor of a *P. pastoris* clone transformed with pPICZα*ChU-B. Fermentation comprised four steps: batch phase for 22 h, glycerol phase for 5 h, transition phase for 5 h to adapt the culture to grow on methanol, induction phase for 136 h. Black circles, laccase activity at the induction phase; black triangles, wet biomass; white squares, extracellular protein concentration.

To test ChU-B expression levels in *P. pastoris*, four different constructs were built, including native and evolved α-factor prepro-leaders in combination with two expression vectors: pPICZαA under the control of the methanol inducible alcohol oxidase promoter (P_AOX1_) and pGAPZαA under the control of the constitutive glyceraldehyde 3-phosphate dehydrogenase promoter (P_GAP_) (Figure 2). Transformed clones were pre-screened for laccase expression on agar plates supplemented with ABTS, resulting in all four cases in a green halo around the colonies due to substrate oxidation by laccase. The apparent most active clones were further subjected to microtiter fermentations (in 96 deep-well plates). Of this set of experiments, P_AOX1_ clones showed the highest ABTS-activity and they were subjected to small scale fed-batch fermentation (500 mL bioreactor, Figure 3A, B). Laccase activity was c.a. 1.7-fold higher for the construct containing the evolved prepro-leader (*i.e.* 580 ABTS-U/L *vs.* 990 ABTS-U/L for the laccase with the original and the mutated α-factor signal sequence, respectively). Accordingly, production of the construct with the evolved prepro-leader was scaled up in a 20-L fermentation. The maximum volumetric activity was reached after 151 h (3220 ABTS-U/L). Cultivation was not stopped at this time since wet biomass was still increasing and we could expect higher amounts of enzyme to be secreted. Unfortunately, laccase activity diminished to 2830 ABTS-U/L at harvesting time (165 h), an effect that may be ascribed to proteolytic degradation by released intracellular proteases, Figure 3C [32]. Under these conditions, the final laccase production was 43 mg/L. This was 5.4-fold higher than that obtained in shake-flask cultures of *S. cerevisiae* (8 mg/L); the latter cannot yield the high cell density levels of *P. pastoris*[32], which precludes its use in bioreactor [28]. Compared to other basidiomycete laccases secreted by *P. pastoris*, the ChU-B secretion was 9-, 5- and 2.5-fold higher than those of laccases from *Pleurotus sajor-caju*, *Pycnoporus cinnabarinus* and *Trametes trogii*, respectively, and very similar to that of the *Trametes* sp. AH28-2 laccase. The production yields achieved with the laccase from *Trametes* sp. 420 and the ascomycete *Botrytis aclada* laccase were much higher (Table 1).

### Biochemical characterization

#### Glycosylation and thermostability

**Figure 4:**
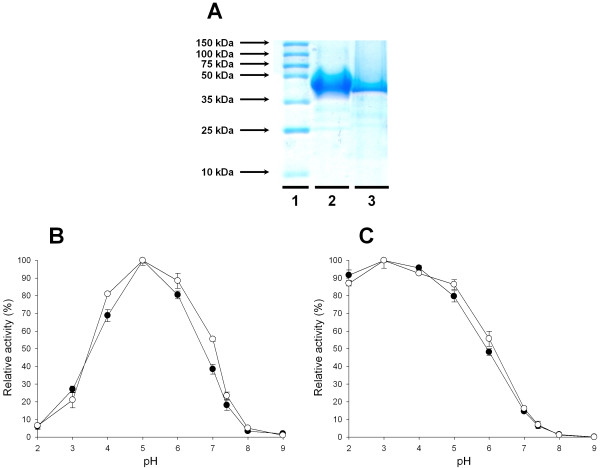
**Biochemical characterization of ChU-B mutant.** (**A**) SDS-PAGE: Lane 1, protein standards; lane 2, ChU-B from *P. pastoris*; lane 3, ChU-B from *S. cerevisiae*. Samples were resolved on a 12% SDS-polyacrylamide gel and stained with Coomassie Brilliant Blue. (**B** and **C**) pH activity profiles. Black circles, ChU-B from *P. pastoris*; white circles, ChU-B from *S. cerevisiae*. Activities were measured in 100 mM Britton and Robinson buffer in the pH range from 3.0 to 9.0 with 3 mM DMP (**B**) or 3 mM ABTS (**C**) as the reducing substrates. Relative activity (in %) was calculated with respect to the activity at the optimum pH and each point, including the standard deviation, is the average of three independent experiments.

The ChU-B laccase produced in *P. pastoris* was purified by three chromatographic steps resulting in a homogeneous sample, which was compared with the purified counterpart from *S. cerevisiae*[31]. The molecular mass deduced from SDS-PAGE was ~60 kDa for the enzyme secreted by *P. pastoris* and *S. cerevisiae*, Figure 4A. The MALDI-TOF (Matrix-Assisted Laser Desorption and Ionization-Time Of Flight) mass spectrometry analysis allowed a more accurate estimation of molecular masses (62541 Da and 60310 Da for the laccase from *P. pastoris* and *S. cerevisiae*, respectively). From the molecular mass determined using the amino acid composition (53939 Da), glycosylation patterns of 16% and 12% for the laccase from *P. pastoris* and *S. cerevisiae* were calculated (Table 2). Unlike *S. cerevisiae*, whose tendency to add in high extent mannose moieties at the Golgi compartment led to hyper-glycosylated heterologous proteins, *P. pastoris* is known to introduce outer sugar chains to a lesser extent (being more appropriate than *S. cerevisiae* to produce, for example, proteins for crystallization studies) [13]. These results address a longer residence time at the Golgi of ChU-B mutant in *P. pastoris* than in *S. cerevisiae*. Comparing our data with other HRPLs expressed in *P. pastoris,* the degree of glycosylation is similar to that of the highly related *T. trogii* laccase (97% of sequence identity with ChU-B [33]) but much lower than that of the hyper-glycosylated *P. cinnabarinus* laccase (with ~75% of sequence identity with ChU-B laccase), which probably has to face several bottlenecks during exocytosis (with glycosylation degrees of 36% and ~50% in *P. pastoris* and *S. cerevisiae*, respectively [22,29]).

**Table 2:** **Biochemical characteristics of ChU-B mutant produced in *P. pastoris *and *S. cerevisiae***

**Feature**	**ChU-B from*P. pastoris***	**ChU-B from*S. cerevisiae***
Expression levels (mg/L)	43	8
MW^1^	53,939	53,939
MW^2^	62,541	60,310
Glycosylation (%)	16	12
N-terminal end	**ETEAEF**SIGP	**ETEAEF**SIGP
Optimum pH (with ABTS)	4	4
Optimum pH (with DMP)	5	5
T_50_ (°C)	57.9	63.7

^1^Calculated from the amino acid composition (http://web.expasy.org/compute_pi/). ^2^Determined by MALDI-TOF mass spectrometry. The extra N-terminal extension is highlighted in bold.

Kinetic thermostability was determined by measuring the T_50_ (the temperature at which the enzyme retains 50% of its activity after ten minutes of incubation). In spite of the fact that hyper-glycosylation is generally reported to confer higher thermostability [34], the T_50_ of the laccase variant produced in *P. pastoris* was ~6°C behind its counterpart from *S. cerevisiae*, Table 2. Only the careful examination of thermodynamic stability could give us new clues about whether the laccase overglycosylation in *P. pastoris* is affecting the protein folding and stability.

#### N-terminal end

We recently reported an extra N-terminal extension of six amino acids in our evolved laccase, as consequence of an alternative processing at the Golgi compartment. It was concluded that this extra tail was beneficial for secretion without jeopardizing the biochemical laccase properties [35]. In order to know whether similar processing takes place in *P. pastoris*, ChU-B was subjected to end-terminal sequencing. Indeed, the same N-terminal extension ETEAEF was detected in the mature protein revealing the lack of sufficient amount of *STE13* protease in *P. pastoris* for a correct cleavage of the α-factor pro-leader. Our results are in good agreement with several studies in which *STE13* was not capable of processing the high levels of α-factor prepro-leaders fusion genes, resulting in an extra N-terminal tail linked to the mature protein [13,35,36].

#### Evolved properties of ChU-B laccase

##### pH activity profiles

HRPLs are fully inactive at neutral or basic pHs due to a reversible OH^-^ inhibition process. One of the most remarkable improvements of ChU-B mutant after directed evolution was the shift in the pH activity profile towards the neutral-alkaline side (pH of human blood is around 7.4). ChU-B produced by *S. cerevisiae* retained ~20% and ~10% of its initial activity at physiological pH with DMP and ABTS as substrates, respectively, whereas the activity of parent type (OB-1 mutant) at this pH was negligible [31]. Almost identical pH activity shapes (with the same optimal pH values at 5.0 for DMP and 4.0 for ABTS) were detected with independency of the producing yeast indicating that this important acquired feature was also shown by the mutant expressed in *P. pastoris* (Figure 4B, C). Even though the pH profile was shifted (including a change in the optimum pH for DMP from 4.0 to 5.0), as occurs for the rest of fungal laccases a bell shaped profile was observed for the phenolic substrate DMP, which is the result of two opposite effects: (i) activation in the acidic range due to higher redox potential difference between the phenol and the T1 Cu (the redox potential of phenols decreases when pH increases while the redox potential of the laccase hardly varies) and (ii) inactivation at alkaline pH due to the accumulation of OH^-^, which bind to the T2 site interrupting the internal electron transfer from the T1 to the T2/T3 centers [37]. Concerning the non-phenolic substrate ABTS, the pH activity profile showed the expected monotonic shape as the oxidation of this compound does not include proton exchange and the only effect involved is the inhibition by OH^-^.

##### Inhibition by halides

**Figure 5:**
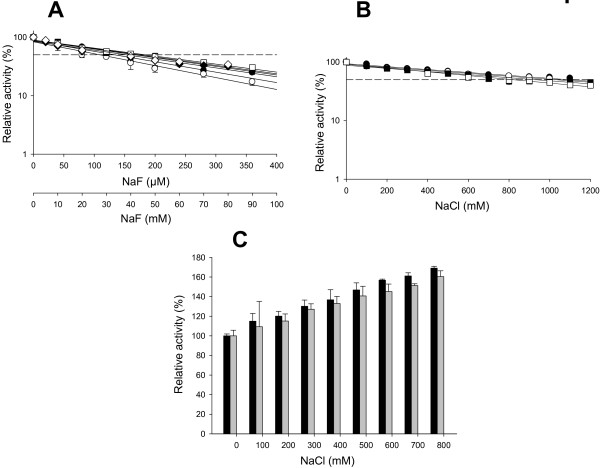
**Inhibition of ChU-B by halides.** (**A**) NaF inhibition with (i) ABTS at pH 4.0 (X-axis in μM; black circles, ChU-B from *P. pastoris*; white circles, ChU-B from *S. cerevisiae*); (ii) DMP at pH 5.0 (X-axis in mM; black squares, ChU-B from *P. pastoris*; white squares, ChU-B from *S. cerevisiae*; (iii) ABTS at pH 7.4 (X-axis in mM; black diamonds, ChU-B from *P. pastoris*; white diamonds, ChU-B from *S. cerevisiae*). (**B**) NaCl inhibition with (i) ABTS at pH 4.0 (black circles, ChU-B from *P. pastoris*; white circles, ChU-B from *S. cerevisiae*); (ii) DMP at pH 5.0 (black squares, ChU-B from *P. pastoris*; white squares, ChU-B from *S. cerevisiae*). (**C**) NaCl inhibition at physiological pH (7.4) with ABTS as substrate (black bars, ChU-B from *P. pastoris*; grey bars, ChU-B from *S. cerevisiae*).

HRPLs are strongly inhibited by the presence of modest concentrations of halides (Cl^-^, Br^-^, F^-^ but not I^-^) [38]. Therefore, the use of HRPLs in miniature biofuel cells operative in mammal physiological fluids (with ~150 mM NaCl) is limited, on the one hand by the negligible activity at neutral-alkaline pH, and on the other, by the low laccase tolerance against Cl^-^. The ChU-B mutant greatly surpassed the halide inhibition by directed evolution and this important property was checked in the variant expressed by *P. pastoris*. The I_50_ values (the concentration of halide at which the enzyme keeps 50% of its initial activity) were determined at acidic and physiological pH using ABTS and DMP as substrates. Whilst the I_50_Cl^-^ of parent type (the OB-1 mutant) was 176 mM and 208 mM for ABTS and DMP at acidic pH, respectively, these values were risen up in the ChU-B variant from *S. cerevisiae* to 1025 mM and 818 mM for these substrates. Additionally, a slight increase in the I_50_F^-^ value for both substrates at acidic pH was also observed (parent type: 70 μM and 167 μM for ABTS and DMP, respectively; ChU-B mutant from *S. cerevisiae*: 109 μM and 183 μM for ABTS and DMP, respectively) [31]. These improved I_50_F^-^ and I_50_Cl^-^ were maintained in the mutant expressed in *P. pastoris* being similar in both yeasts (*i.e.* I_50_Cl^-^ ~1000 mM and I_50_F^-^ above 100 μM both for ABTS and DMP at acidic pH), Table 3, Figure 5A, B. Since the smaller the ionic diameter of the halide the easier the access to the T2/T3 trinuclear copper cluster [39], an inhibition potency F^-^>Cl^-^ was observed with independence of the substrate tested, Table 3, Figure 5A, B. When halide inhibition was measured at physiological pH, the enzyme expressed in both yeasts showed I_50_F^-^ which rose from the μM range at acid pH to the mM range at blood pH, Figure 5A. Moreover, laccase activity was not affected by increasing concentrations of Cl^-^ (Figure 5C). This data is consistent with the described effect by which the halide inhibition of laccase activity is weaker at alkaline pH values. Under such conditions, the presence of a deprotonated water molecule coordinating the T2 Cu results in a competition with the halide for binding to the T2 site [37,40,41].

**Table 3:** **I**_**50 **_**values (in mM) of sodium halides for ChU-B mutant produced in *P. pastoris *and *S. cerevisiae***

**Inhibitor**	**Substrate**	**pH**	**ChU-B from*P. pastoris*I**_**50**_**(mM)**	**ChU-B from *S. cerevisiae *I**_**50**_**(mM)**
NaF	ABTS	4.0	0.134	0.109
	ABTS	7.4 (blood buffer)	40	42
	DMP	5.0	0.174	0.183
NaCl	ABTS	4.0	1106	1025
	ABTS	7.4	n.m.	n.m.
	DMP	5.0	931	818

n.m. non-measurable. Inhibition studies were performed at the optimum pH activity value for each substrate tested (4.0 and 5.0 for ABTS and DMP, respectively) and at physiological pH (7.4) with ABTS as reducing substrate.

#### Kinetics

**Figure 6:**
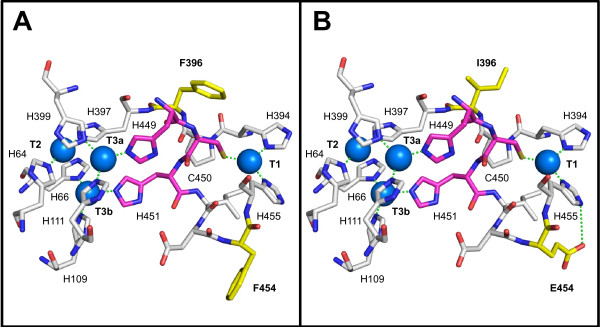
**Location of the two mutations responsible for blood tolerance in the ChU-B mutant (B) compared with the corresponding residues in the parental type OB-1 (A).** The F396I and F454E mutations are shown in yellow sticks and copper ions are depicted as blue spheres. The three residues in charge of the internal electron transfer from T1 Cu to T2/T3 cluster are displayed as magenta sticks. Residues involved in the first coordination sphere of the catalytic coppers and their interactions (green dashes) are also represented. The 3D-structure model is based on the crystal structure of the *Trametes trogii* laccase (97% identity, PDB: 2HRG) [33].

Kinetics parameters were measured for phenolic and non-phenolic substrates at optimum and physiological pH (Table 4). The *K*_m_ for ABTS and DMP was similar for the laccase produced either by *S. cerevisiae* or *P. pastoris*. By contrast, the *k*_cat_ values for the two substrates were around 2.7 and 4.8-fold higher for the laccase from *S. cerevisiae* than those of the laccase from *P. pastoris*. Possibly, the detected glycosylation differences between both laccases are in part responsible for this effect. Further crystallization studies along with computational analysis would be important to clarify the differences in *k*_cat_ values and thermostabilities [42]. When comparing kinetics with the original parent type expressed in *S. cerevisiae*[28], the *K*_m_ at acidic pH was increased around 4- and 14-fold whereas the *k*_cat_ was ~3.5- and 7-fold lower than those of the parental type, for ABTS and DMP respectively. Mutations F396I and F454E, both located at the second coordination sphere of the T1 Cu, enabled the enzyme to be active under physiological conditions albeit at the cost of catalytic efficiency (Figure 6). The activity of ChU-B from *P. pastoris* in physiological fluids was determined by measuring the oxygen consumption in human plasma and blood. Comparable responses for both human fluids were obtained (31 ± 7 and 27 ± 1 min^-1^ for blood and plasma, respectively). Since for the application of this enzyme in 3D-nanodevices working in physiological conditions, the laccase is directly connected to the cathode of a biofuel cell, the reducing substrates are replaced by a direct electronic current from the anode, which is the rate limiting step in the catalytic mechanism. In fact, ChU-B is functional in blood because of the slowed down kinetics. As we have recently reported, the modification of the second coordination sphere of the T1 Cu comes at the cost of reducing the activity at acidic values, which simultaneously compensates for T2 Cu inhibition activating ChU-B in the presence of halides and OH^-^[31].

**Table 4:** **Steady-state kinetic parameters of ChU-B mutant expressed in *P. pastoris *and *S. cerevisiae***

**Substrate**	**pH**	**ChU-B from*P. pastoris***			**ChU-B from*S. cerevisiae***			**(Parental type, OB-1mutant**^**a**^**)**		
		**K**_**m**_**(mM)**	**k**_**cat**_**(s**^**-1**^**)**	**k**_**cat**_**/K**_**m**_**(mM**^**-1**^**s**^**-1**^**)**	**K**_**m**_**(mM)**	**k**_**cat**_**(s**^**-1**^**)**	**k**_**cat**_**/K**_**m**_**(mM**^**-1**^**s**^**-1**^**)**	**K**_**m**_**(mM)**	**k**_**cat**_**(s**^**-1**^**)**	**k**_**cat**_**/K**_**m**_**(mM**^**-1**^**s**^**-1**^**)**
ABTS	3.0	0.024 ± 0.002	22.3 ± 0.5	929	0.023 ± 0.001	57.0 ± 0.9	2478	0.0063 ± 0.0009	200 ± 7	31746
	7.4	0.26 ± 0.01	1.13 ± 0.02	4.35	0.32 ± 0.02	3.09 ± 0.07	9.66	n.d.	n.d.	n.d.
DMP	5.0	1.91 ± 0.05	5.65 ± 0.05	2.96	2.0 ± 0.2	19.5 ± 0.5	9.75	0.14 ± 0.02	134 ± 5	957
	7.4	0.23 ± 0.03	0.41 ± 0.02	1.78	0.22 ± 0.03	1.95 ± 0.05	8.86	n.d.	n.d.	n.d.

^a^ Data from Maté *et al.*, 2010. n.d. not determined.

## Conclusions

The blood tolerant laccase engineered by laboratory evolution in *S. cerevisiae* is easily secreted in *P. pastoris* with higher production yields whilst maintaining its evolved properties in terms of halide tolerance and pH activity profiles. These results support the use of *S. cerevisiae* as the preferred host to evolve ligninolytic enzymes and *P. pastoris* to over-express them for different purposes. Indeed, the application of this tandem-yeast evolution/expression system can be extended from laccases to other ligninolytic oxidoreductases (lignin-, manganese- and versatile-peroxidases, aromatic peroxygenases, aryl alcohol oxidases) whose engineering for challenging biocatalytic applications are currently pursued by many research groups.

## Methods

### Strains and chemicals

The *P. pastoris* expression vectors pPICZαA and pGAPZαA, the *Escherichia coli* strain DH5α, the *P. pastoris* strain X-33 and the antibiotic Zeocin were purchased from Invitrogen (Carlsbad, CA, USA). Restriction endonucleases, the Rapid DNA Ligation Kit, containing T4 DNA ligase, and the shrimp alkaline phosphatase were obtained from Fermentas (Burlington, Canada). Nucleic acid amplifications were done employing Phusion High-Fidelity DNA Polymerase from New England Biolabs (Beverly, USA), dNTP mixture from Thermo Fisher Scientific (Waltham, MA, USA) and oligonucleotide primers from VBC Biotech (Vienna, Austria). The Illustra GFX PCR DNA and gel band purification kit was obtained from GE Healthcare (Buckinghamshire, UK). All chemicals and media components were of the highest purity available.

### Laccase functional expression in *P. pastoris*

#### Laccase constructs for P. pastoris

A 1.5-kDa DNA fragment containing the coding region of the ChU-B mutant laccase gene was cloned with the original and the mutated α-factor prepro-leader from *S. cerevisiae* into the expression vectors pPICZαA and pGAPZαA. The vector pJRoC30α*ChU-B, resulting from a previous directed evolution experiment [31], was used to amplify the laccase gene without the evolved α-factor signal peptide with the primers 5PM1EcoR1 (5’-AAGAATTCAGCATTGGGCCAGTCGCAG-3’) and 3PM1Xba1 (5´-AGGTCTAGATTACTGGTCGTCAGGCGAG-3´), which included targets for *Eco*RI and *Xba*I restriction enzymes, respectively (in bold). The laccase gene fused to the evolved α-factor signal sequence was amplified using the primers 5ALPHABst1 (5´-ATTTCGAAACGATGAGATTTCCTTCAATTTTTACTGC-3´), which included the *Bst*BI target (in bold), and 3PM1Xba1. PCR reactions were performed using a GeneAmp PCR System 2700 thermocycler (Applied Biosystems, Foster City, CA, USA) in a final volume of 25 μL containing 0.6 μM of each primer, 2 ng template, 800 μM dNTPs (200 μM each), 3% dimethyl sulfoxide (DMSO), 1.5 mM MgCl_2_ and 0.5 U of Phusion polymerase. The PCR conditions were 98°C for 30 sec (1 cycle); 98°C for 10 sec, 62°C for 20 sec, 72°C for 45 sec (30 cycles); and 72°C for 7 min (1 cycle). The PCR products were purified using the Illustra GFX PCR DNA and gel band purification kit and then digested with the restriction enzymes *Bst*BI and *Xba*I -in the case of the fusion gene- or *Eco*RI and *Xba*I -in the case of the gene encoding the mature protein- at 37°C for 3 h. The pPICZαA and pGAPZαA vectors were equally treated and then their 5’ and 3’ ends were dephosphorylated using shrimp alkaline phosphatase at 37°C for 1 h. The PCR product and the linearized vector were ligated with T4 DNA ligase at room temperature for 30 min. After transformation of the constructs into chemically competent *E. coli* DH5α cells, the plasmids were proliferated, linearized with the restriction enzyme *Sac*I at 37°C for 1 h and transformed into electro-competent *P. pastoris* X-33 cells. Electro-competent *Pichia* cells were prepared and transformed following the condensed protocol of Lin-Cereghino *et al.*[43]. Transformants were grown on YPD plates (10 g/L yeast extract, 20 g/L peptone, 4 g/L glucose and 15 g/L agar) containing 25 mg/L zeocin and screened on indicator agar plates with BMM agar (100 mM potassium phosphate buffer pH 6.0, 3.5 g/L yeast nitrogen base without amino acids nor ammonium sulfate, 10 g/L ammonium sulfate, 400 μg/L biotin, 0.5% methanol, 20 g/L agar) containing 0.2 mM ABTS and 0.1 mM CuSO_4_.

### Small scale fed-batch fermentation

*P. pastoris* clone harbouring the ChU-B mutant with the original (α) and/or the mutated (α*) -factor signal peptide under control of the AOX1 promoter was cultivated in a 500-mL Multifors bioreactor (Infors HT, Bottmingen, CH) with a starting volume of 300 mL basal salts medium (26.7 mL/L 85% phosphoric acid, 0.93 g/L CaSO_4_·2H_2_O, 14.9 g/L MgSO_4_·7H_2_O, 18.2 g/L K_2_SO_4_, 4.13 g/L KOH; 40 g/L glycerol). After sterilization, the medium was supplemented with 4.35 mL/L PTM_1_ trace salts (as described by Invitrogen), 100 μL Antifoam 204 (Sigma, St. Louis, MO, USA) and 0.1 mM CuSO_4_. The pH of the medium was adjusted to pH 5.0 with 28% ammonium hydroxide and maintained at this value throughout the whole fermentation process. The fermentations were started by adding 25 mL of preculture grown on YPD medium in 250-mL baffled shake flasks at 125 rpm and 30°C overnight. The cultivations were performed according to the *Pichia* Fermentation Process Guidelines of Invitrogen with some modifications. The batch was run at 30°C, 500 rpm and an air flow of 0.2 L/min. After depletion of the glycerol in the batch medium, the fed-batch phase was started with a feed of 50% (w/v) glycerol containing 12 mL/L PTM1 trace salts for 5 h to increase the cell biomass under limiting conditions. For induction, the temperature was reduced to 25°C and the feed was switched to 100% methanol with 12 mL/L PTM_1_ trace salts at an initial feed rate of 0.6 mL/h until the culture was fully adapted to methanol. Subsequently the feed rate was adjusted to keep the oxygen saturation constant at 4% at a constant air supply of 2 L/min and a stirrer speed of 800 rpm. Samples were taken regularly and clarified by centrifugation. The wet biomass was measured by weighing centrifuged tubes containing culture samples after removing the supernatant. The soluble protein concentration was quantified using the Bio-Rad Protein Assay (Bio-Rad, CA, USA), with bovine serum albumin as standard. The volumetric activity was assayed spectrophotometrically using ABTS (ϵ_ABTS•+, 418nm_=36,000 M^-1^ cm^-1^) as substrate. The reaction was followed for 5 min at room temperature in a Lambda 35 UV/Vis spectrophotometer (Perkin Elmer). The ABTS-based assay contained 3 mM ABTS final concentration in 100 mM sodium acetate buffer pH 4.0.

### Large scale fed-batch fermentation

The α*-ChU-B mutant under control of the AOX1 promoter (pPICZα*ChU-B construct) was large-scale produced in *P. pastoris* using a 42-L autoclavable stainless steel bioreactor (Applikon Biotechnology, Schiedam*,* The Netherlands) filled with 10 L of basal salts medium. After sterilization, 4.35 mL/L PTM_1_ trace salts and 2 mL Antifoam 204 were added to the medium. Furthermore, the pH was set to pH 5.0 with 28% ammonium hydroxide, keeping it at this value throughout the entire process. The fermentation was started by adding 1 L of *P. pastoris* preculture grown on YPD medium in several 1-L baffled shake flasks at 200 rpm and 30°C overnight. According to the *Pichia* Fermentation Process Guidelines aforementioned, the batch was run at 30°C and 600 rpm, keeping the dissolved oxygen (DO) concentration above 4%. Once all the glycerol was consumed from the batch growth phase, the glycerol fed phase was started with a feed of 50% (w/v) glycerol containing 12mL/L PTM_1_ trace salts for 5 h to increase the biomass. Afterwards, 0.5% (v/v) methanol with 12 mL/L PTM_1_ trace salts and 0.1 mM CuSO_4_ were injected aseptically into the fermenter. From this time on, the temperature was set to 25°C and the stirrer speed to 750 rpm. After 5 h of transition phase, the feed was switched to 100% methanol containing 12 mL/L PTM_1_ trace salts and it was regulated to keep the DO concentration between 1 and 3%. Samples were taken regularly and wet biomass, protein concentration and laccase activity were determined as mentioned above.

### Purification of the laccase produced in *P. pastoris*

The culture broth of ChU-B mutant containing the *P. pastoris* cells was clarified by centrifugation at 6000 rpm for 20 min at 4°C (Sorvall Evolution RC Superspeed Centrifuge, Thermo Fisher Scientific) and solid ammonium sulphate was slowly added to the supernatant to 30% saturation at 4°C. The suspension was centrifuged at 6000 rpm for 30 min at 4°C to discard the precipitated protein (without laccase activity). Then, the supernatant-containing laccase activity was applied to a 750-mL PHE sepharose 6 FF column (chromatographic equipment and materials from GE Healthcare) equilibrated with 50 mM sodium acetate buffer pH 5.0 containing 30% saturation ammonium sulphate. Proteins were eluted within a linear gradient from 30 to 0% ammonium sulphate at a flow rate of 20 L/min for 2 h. Fractions with laccase activity were pooled, dialyzed and concentrated in 20 mM Bis-Tris HCl buffer pH 6.5 (buffer A) using a hollow fiber cross-flow module (Microza UF module SLP-1053, 10 kDa cut-off, Pall Corporation, Port Washington, NY, USA). The sample was loaded onto a 19-mL Mono Q column, previously equilibrated with buffer A. Proteins were eluted with a linear gradient from 0 to 0.4 M of NaCl at a flow rate of 2 mL/min for 1 h. Active fractions were pooled and applied to a 70 mL PHE source column. Laccase was eluted with a linear gradient from 15 to 0% ammonium sulphate at a flow rate of 1 mL/min for 6 h. The fractions with laccase activity were pooled, dialyzed against buffer A, concentrated and stored at 4°C.

### Production and purification of the laccase expressed in *S. cerevisiae*

The ChU-B mutant was expressed in the protease deficient *Saccharomyces cerevisiae* strain BJ5465 (LGC Promochem, Barcelona, Spain) and purified to homogeneity following the protocol reported in a former work [28].

### Kinetic thermostability (T_50_ determination)

The thermostability of the different laccase samples was estimated by assessing their T_50_ values using 96/384 well gradient thermocyclers. Appropriate laccase dilutions were prepared, such that 20 μL aliquots produced a linear response in the kinetic mode. Subsequently, 50 μL samples were assessed at each point in the gradient scale and a temperature gradient profile ranging from 35 to 90°C was established as follows (in °C): 35.0, 36.7, 39.8, 44.2, 50.2, 54.9, 58.0, 60.0, 61.1, 63.0, 65.6, 69.2, 72.1, 73.9, 75.0, 76.2, 78.0, 80.7, 84.3, 87.1, 89.0 and 90.0. After 10 min of incubation, the samples were chilled on ice for 10 min and further incubated at room temperature for 5 min. Next, 20 μL of samples were subjected to the same ABTS-based colorimetric assay described above. Thermostability values were deduced from the ratio between the residual activities incubated at different temperature points and the initial activity at room temperature.

### pH activity profiles

Appropriate laccase dilutions were prepared in such a way that 10 μL aliquots produced a linear response in the kinetic mode. Plates containing 10 μL of laccase samples and 180 μl of 100 mM Britton and Robinson buffer were prepared at pH values of 2.0, 3.0, 4.0, 5.0, 6.0, 7.0, 8.0 and 9.0. The assay commenced when 10 μL of 60 mM ABTS or DMP was added to each well to give a final substrate concentration of 3 mM. The activities were measured in triplicate in kinetic mode and the relative activity (in %) is based on the maximum activity for each variant in the assay.

### Halide inhibition (I_50_ determination)

The inhibitory effect of fluoride and chloride was measured using two laccase substrates (ABTS and DMP) at their corresponding optimal pH activity values (in 100 mM sodium acetate buffer (pH 4.0) for ABTS and 100 mM sodium tartrate buffer (pH 5.0) for DMP, as well as at physiological pH (in 100 mM sodium phosphate buffer, pH 7.4). Inhibition was determined by the I_50_ value (the halide concentration at which only 50% of the initial laccase activity is retained), as the complexity of the plots complicated the extraction of the inhibition constant (*K*_*i*_). The assay mixture contained 2.4 mM ABTS or DMP, halide (concentrations ranging from 0 to 1100 mM) and purified laccase (0.2 and 1.7 nM for ABTS and DMP, respectively). Each data point represents the mean value determined in at least three independent experiments.

### Kinetics parameters

As previously reported [28], steady-state enzyme kinetics were determined using the following extinction coefficients: ABTS, ϵ_418_ = 36000 M^-1^ cm^-1^; DMP, ϵ_469_ = 27500 M^-1^ cm^-1^ (relative to substrate concentration). To calculate the values of *K*_m_ and *k*_cat,_ the average *v*_max_ was represented versus substrate concentration and fitted to a single rectangular hyperbola function in SigmaPlot 10.0, where parameter *a* equales *k*_cat_ and parameter *b* equals *K*_m_.

### Determination of laccase activity in human plasma and blood

Human blood was collected in BD Vacutainer® blood collection tubes (Plymoth, UK). Blood samples were centrifuged for 10 min at 3000 rpm to obtain human plasma, discarding the pellet after having extracted the supernatant. Both plasma and blood were supplemented with 10 mM ascorbic acid as laccase substrate and the pH adjusted to 7.4. The activity of the ChU-B mutant in both physiological fluids was determined by measuring oxygen consumption in solution with a Clark electrode. These experiments were performed using the Oxygraph system (Hansatech Instruments, King´s Lynn, UK).

### MALDI-TOF analysis

Matrix Assisted Laser Desorption and Ionization-Time Of Flight (MALDI-TOF) experiments were performed on an Autoflex III MALDI-TOF-TOF instrument (Bruker Daltonics, Bremen, Germany) with a smartbeam laser. The spectra were acquired at a laser power just above the ionization threshold, and the samples were analysed in the positive ion detection and delayed extraction linear mode. Typically, 1000 laser shots were summed into a single mass spectrum. External calibration was performed, using BSA from Bruker, over a range of 30000–70000 Da. The 2,5-dihydroxy-acetophenone (2,5-DHAP) matrix solution was prepared by dissolving 7.6 mg (50 μmol) in 375 μL ethanol, to which 125 μL of 80 mM diammonium hydrogen citrate aqueous solution was added. For sample preparation, 2.0 μL of purified enzyme was diluted with 2.0 μL of 2% trifluoro acetic acid aqueous solution and 2.0 μL of matrix solution. A volume of 1.0 μL of this mixture was spotted onto the stainless steel target and allowed to dry at room temperature.

### N-terminal analysis

Purified laccases were resolved by SDS-PAGE and the proteins transferred to polyvinylidene difluoride (PVDF) membranes. The PVDF membranes were stained with Coomassie Brilliant Blue R-250, after which the enzyme bands were cut out and processed for N-terminal amino acid sequencing on a precise sequencer at the Core facilities of the Helmholtz Centre for Infection Research (HZI; Braunschweig, Germany).

### Protein modeling

The 3D-structure models of the PM1 mutant laccases are based on the crystal structure of the *Trametes trogii* laccase (PDB: 2HRG, 97% sequence identity with the PM1 laccase) [33]. The protein models were generated and analyzed as formerly reported [28].

## Abbreviations

PAOX1: Alcohol oxidase 1 promoter; ABTS: 2,2'-azino-bis(3-ethylbenzothiazoline-6-sulphonic acid); DMP: 2,6-dimethoxyphenol; DO: Dissolved oxygen; PGAP: Glyceraldehyde 3-dehydrogenase promoter; HRPL: High-redox potential laccase; I50: The halide concentration which causes 50% decrease of the initial laccase activity; MALDI-TOF: Matrix-assisted laser desorption and ionization-time of flight; SDS-PAGE: Sodium dodecyl sulfate-polyacrylamide gel electrophoresis; T50: The temperature at which the enzyme loses 50% of its initial activity following incubation for 10 minutes

## Competing interests

The authors declare that they have no competing interests.

## Authors’ contributions

DMM carried out the production and purification of ChU-B mutant both in *P. pastoris* and *S. cerevisiae*. DGP collaborated in the purification of ChU-B mutant expressed in *S. cerevisiae*. DMM and DGP did the biochemical characterization of ChU-B produced in both yeasts. RK performed the vector constructions for laccase expression in *P. pastoris*. DMM wrote the first draft of the manuscript. RL co-supervised the project and revised the manuscript. MA coordinated the project, supervised its development and wrote the final manuscript, which was read and approved by all authors.
